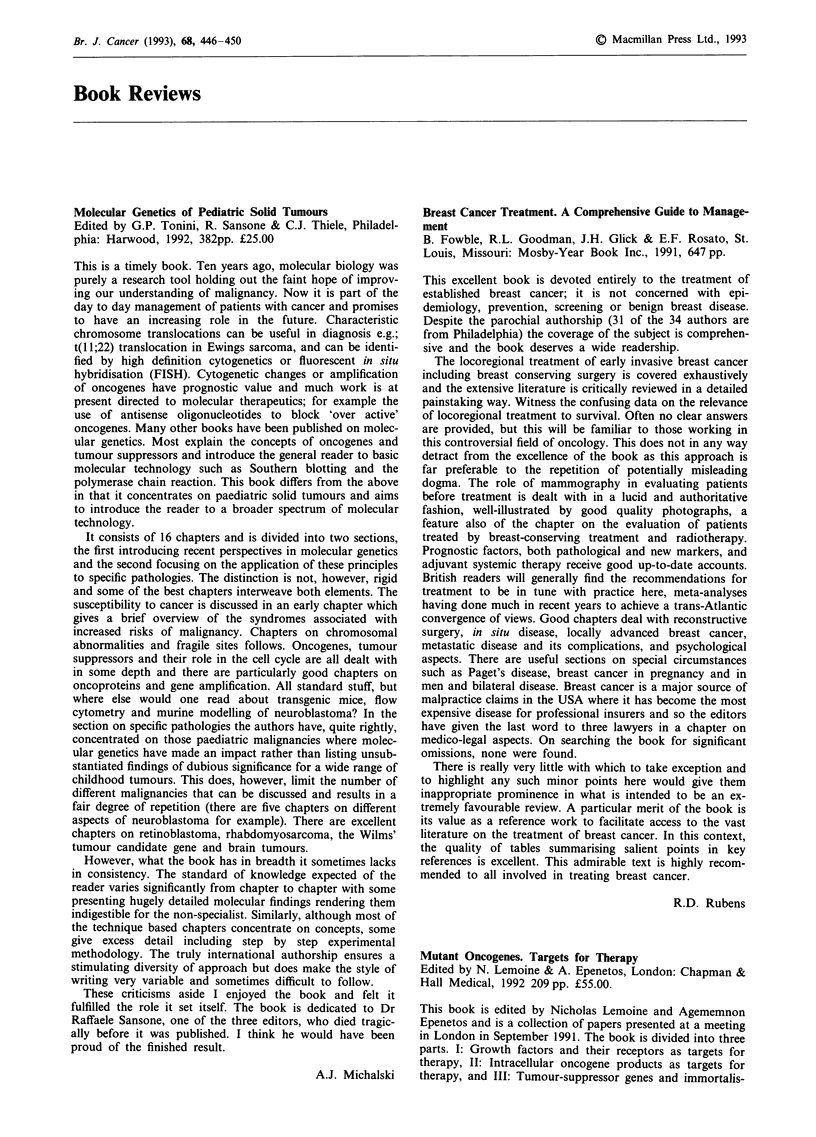# Breast Cancer Treatment. A Comprehensive Guide to Management

**Published:** 1993-08

**Authors:** R.D. Rubens


**Breast Cancer Treatment. A Comprehensive Guide to Management**
B. Fowble, R.L. Goodman, J.H. Glick & E.F. Rosato, St. Louis, Missouri: Mosby-Year Book Inc., 1991, 647 pp.

This excellent book is devoted entirely to the treatment of established breast cancer; it is not concerned with epidemiology, prevention, screening or benign breast disease. Despite the parochial authorship (31 of the 34 authors are from Philadelphia) the coverage of the subject is comprehensive and the book deserves a wide readership.

The locoregional treatment of early invasive breast cancer including breast conserving surgery is covered exhaustively and the extensive literature is critically reviewed in a detailed painstaking way. Witness the confusing data on the relevance of locoregional treatment to survival. Often no clear answers are provided, but this will be familiar to those working in this controversial field of oncology. This does not in any way detract from the excellence of the book as this approach is far preferable to the repetition of potentially misleading dogma. The role of mammography in evaluating patients before treatment is dealt with in a lucid and authoritative fashion, well-illustrated by good quality photographs, a feature also of the chapter on the evaluation of patients treated by breast-conserving treatment and radiotherapy. Prognostic factors, both pathological and new markers, and adjuvant systemic therapy receive good up-to-date accounts. British readers will generally find the recommendations for treatment to be in tune with practice here, meta-analyses having done much in recent years to achieve a trans-Atlantic convergence of views. Good chapters deal with reconstructive surgery, *in situ* disease, locally advanced breast cancer, metastatic disease and its complications, and psychological aspects. There are useful sections on special circumstances such as Paget's disease, breast cancer in pregnancy and in men and bilateral disease. Breast cancer is a major source of malpractice claims in the USA where it has become the most expensive disease for professional insurers and so the editors have given the last word to three lawyers in a chapter on medico-legal aspects. On searching the book for significant omissions, none were found.

There is really very little with which to take exception and to highlight any such minor points here would give them inappropriate prominence in what is intended to be an extremely favourable review. A particular merit of the book is its value as a reference work to facilitate access to the vast literature on the treatment of breast cancer. In this context, the quality of tables summarising salient points in key references is excellent. This admirable text is highly recommended to all involved in treating breast cancer.

R.D. Rubens